# Thiol-Capped Gold Nanoparticles Swell-Encapsulated into Polyurethane as Powerful Antibacterial Surfaces Under Dark and Light Conditions

**DOI:** 10.1038/srep39272

**Published:** 2016-12-16

**Authors:** Thomas J. Macdonald, Ke Wu, Sandeep K. Sehmi, Sacha Noimark, William J. Peveler, Hendrik du Toit, Nicolas H. Voelcker, Elaine Allan, Alexander J. MacRobert, Asterios Gavriilidis, Ivan P. Parkin

**Affiliations:** 1 Department of Chemistry, University College London, 20 Gordon St, London, WC1H 0AJ, United Kingdom; 2 Department of Chemical Engineering, University College London, Torrington Place, London, WC1E 7JE, United Kingdom; 3 ARC Centre of Excellence for Convergent Bio-Nano Science and Technology, Future Industries Institute, University of South Australia, Mawson Lakes, 5095, Australia; 4 Division of Microbial Diseases, UCL Eastman Dental Institute, University College London, 256 Grays Inn Road, London, WC1X 8LD, United Kingdom; 5 UCL Division of Surgery and Interventional Science, Royal Free Campus, Rowland Hill Street, London, NW3 2PF, United Kingdom

## Abstract

A simple procedure to develop antibacterial surfaces using thiol-capped gold nanoparticles (AuNPs) is shown, which effectively kill bacteria under dark and light conditions. The effect of AuNP size and concentration on photo-activated antibacterial surfaces is reported and we show significant size effects, as well as bactericidal activity with crystal violet (CV) coated polyurethane. These materials have been proven to be powerful antibacterial surfaces against both Gram-positive and Gram-negative bacteria. AuNPs of 2, 3 or 5 nm diameter were swell-encapsulated into PU before a coating of CV was applied (known as PU-AuNPs-CV). The antibacterial activity of PU-AuNPs-CV samples was tested against *Staphylococcus aureus* and *Escherichia coli* as representative Gram-positive and Gram-negative bacteria under dark and light conditions. All light conditions in this study simulated a typical white-light hospital environment. This work demonstrates that the antibacterial activity of PU-AuNPs-CV samples and the synergistic enhancement of photoactivity of triarylmethane type dyes is highly dependent on nanoparticle size and concentration. The most powerful PU-AuNPs-CV antibacterial surfaces were achieved using 1.0 mg mL^−1^ swell encapsulation concentrations of 2 nm AuNPs. After two hours, Gram-positive and Gram-negative bacteria were reduced to below the detection limit (>4 log) under dark and light conditions.

Hospital-acquired infections (HAIs) are a severe threat to public health, worsened by the increasing occurrence of multi-drug resistant bacteria^1^,^2^. The Department of Health UK reported that an estimated 25,000 people across the European Union (EU) die each year as a result of HAIs caused by resistant strains of bacteria^3^. Moreover, the associated cost of infections and infectious diseases in the UK is approximately £30 billion each year^3^; a significant burden on financially-stretched healthcare services.

A key way in which bacteria spread in healthcare environments is through patient-healthcare worker-surface contact. This can occur through bench tops, telephones, food trays and implanted medical equipment such as catheters. Current surface disinfection protocols help prevent the build-up of bacteria and decrease the spread of infections. However, this strategy has some disadvantages including cost, difficulties in thorough cleaning and/or disinfection, strongly adherent bacteria remaining on the surface, and the growth of bacterial colonies from residual traces^4^. This cyclic transmission of bacteria can be reduced using antibacterial surfaces to decrease surface bacterial contamination in hospital environments^5^,^6^.

There are many reports of antimicrobial materials for the prevention of bacterial surface contamination, such as antibiotic hydrogels^7^, copper^8^, silicone^9^, and polyamine based polymeric films^10^. These have shown varying antimicrobial efficacies, with limitations including the potential for the development of antimicrobial resistance to incorporated antibiotics, and the build-up of dirt on surfaces, obscuring the inherent antibacterial surface properties. One alternative approach involves the photosensitisation of bacteria *via* reactive oxygen species (ROS) using light-active surfaces such as TiO_2_^11^ or photosensitised dye-modified materials^12^,^13^,^14^. A photosensitisation approach is promising since this form of antibacterial technology can be easily incorporated to polymer surfaces^15^ for applications in healthcare environments such as hospital surfaces and/or medical devices such as catheters. Photosensitised dyes including crystal violet (CV), methylene blue (MB) and toluidine blue O (TBO), in addition to nanoparticles, can be incorporated into medical grade polymers using a simple swell-encapsulation-shrink method^15^,^16^,^17^. Upon illumination, the polymer-immobilised dye molecule is promoted to an excited singlet state, which can then undergo inter-system crossing to the triplet state, after which it can partake in a series of different photochemical reactions. These photochemical reactions yield a range of different ROS *via* a Type I or Type II process; Type I reaction is an electron transfer process, which results in the generation of hydroxyl radicals or superoxides. Type II reaction involves an energy transfer process in which singlet state oxygen is generated^18^. Collectively, the ROS generated in these processes are toxic to bacteria^19^ and due to the non-site specific mode of bacterial attack, the emergence of bacterial resistance to this strategy is unlikely. Moreover, recent research has demonstrated that this antibacterial surface strategy is suitable for both Gram-positive and Gram-negative bacteria representative of those responsible for HAIs^8^,^16^,^17^.

Substantial efforts have been made on the production of nanoparticles where their unique and interesting properties can be tailored by adjusting their particle size and shape^20^,^21^,^22^. Gold nanoparticles (AuNPs) and other gold-based nanomaterials have generally been considered inert, non-toxic and bio-compatible^23^,^24^,^25^ and AuNPs continue to show great potential as therapeutic and medical imaging agents^26^,^27^,^28^,^29^,^30^, AuNPs possess a high surface-to-volume ratio, easily tunable size, and facile surface modification, rendering them versatile platforms for healthcare materials^31^,^32^,^33^. Although AuNPs have no intrinsic bactericidal effects, studies have shown that aqueous 2 nm AuNPs in combination with photosensitised dyes show a synergistic enhancement in the photobactericidal activity of the sample^34^,^35^. This enhancement in photobactericidal activity has been previously shown to be dependent on the size of AuNPs^34^. Time resolved electron paramagnetic resonance (TR-EPR) measurements have shown that small (2 nm) AuNPs generate a ca. 40% increase in MB dye-triplet state production, and increased triplet yields contribute to enhanced ROS generation^19^. Despite this, all previous studies on the incorporation of AuNPs (2 nm and above) in antibacterial surfaces have been done using aqueous AuNPs^34^,^35^. or multi-dye-AuNP combinations in silicone^16^. Multi-dye AuNP combinations involved determining the antibacterial effectiveness of both MB, CV^36^ and their combination^16^. The most effective dye was found to be CV while the combination of CV and MB was also very effective against Gram-positive and Gram-negative bacteria^16^,^36^. An alternative to the approaches described above is to incorporate AuNPs that are stabilized with alkylthiol ligands, rendering them soluble in organic solvents such as toluene. These particles are easy to synthesise and their size is precisely controlled. Furthermore, due to the hydrophobic thiol-capping, the AuNPs are more easily incorporated into polymers during swelling, and this allows for finer control over final particle concentration when compared to aqueous AuNP suspensions. The stabilizing thiol groups may also act as functional ligands, which have been previously shown to provide direct multivalent interactions with biological molecules^37^. This allows nanoparticles, such as AuNPs, to be exploited as self-therapeutic agents against bacteria. Previous work by Li *et al*., has shown thiol-functional ligands on 2 nm core AuNPs can tailor particle surface hydrophobicity which provides a new dimension to antibacterial nanomaterials^38^.

Herein, we demonstrate an enhancement in light and dark antibacterial activity for polyurethane (PU) coated with CV in combination with encapsulated thiol-capped AuNPs. AuNP encapsulated CV coated PU (PU-AuNPs-CV) samples were prepared by a two stage swell-encapsulation process and characterised using transmission electron microscopy (TEM), UV-visible spectroscopy, x-ray photoelectron spectroscopy (XPS), time of flight secondary ion mass spectroscopy (ToF-SIMS), water contact angle measurements and fluorescence microscopy. The bactericidal activity of the encapsulated PU samples was tested against *Staphylococcus aureus* and *Escherichia coli* as representative Gram-positive and Gram-negative bacteria, respectively. The antibacterial properties were studied by investigating different sizes and concentrations of AuNPs in the swelling solutions. All antibacterial tests were conducted with thiol-incorporated PU as a control to ensure no antibacterial activity was coming from the capping agent. The ‘swell-encapsulation-shrink’ technique is a straightforward and scalable approach to target harmful bacteria making this type of antibacterial surface particularly interesting for clinical and/or hospital use.

## Results and Discussion

### Nanoparticle Characterisation

**Figure 1:**
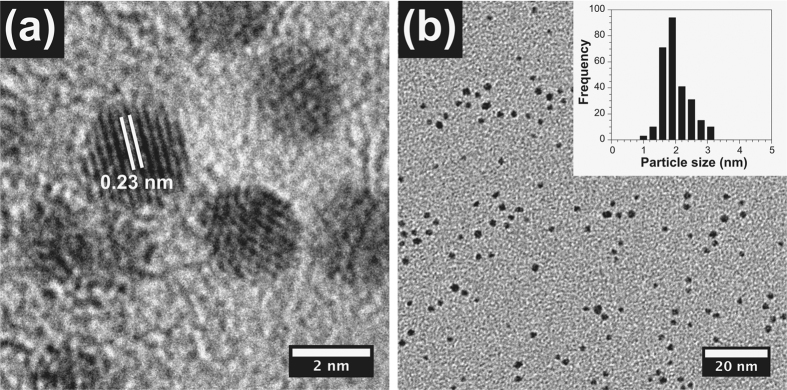
HR-TEM images of (**a**) 2 nm AuNPs lattice fringes, (**b**) 2 nm AuNPs (inset shows histogram for particle count).

Batches of size controlled thiol-capped AuNPs sized between 2–5 nm were synthesised by methods reported previously^39^,^40^,^41^. In a typical synthesis, chloroauric acid (HAuCl_4_) was reduced with sodium borohydride (NaBH_4_) in the presence of 1-dodecanethiol (DDT). The presence of DDT resulted in stabilized thiol-capped AuNPs sized between 2–5 nm, which was controlled by both the amount of reducing, and capping agents. Figure 1(a,b) shows typical HR-TEM images for the smallest and most effective AuNPs with an average particle size of 2.0 nm (s.d. 0.4 nm) and lattice spacing of 0.23 nm (Au 111). HR-TEM for AuNPs of 3.0 nm (s.d. 1.0 nm) and 5.0 nm (s.d. 1.5 nm) can be seen in Supplementary Fig. S1. All AuNPs were non-agglomerated and produced spherical nanoparticles, with no other morphologies observed. The UV-visible absorption spectra for the 3 and 5 nm AuNPs exhibited surface plasmon resonance (SPR) peaks at 517 nm and 532 nm, respectively (Supplementary Fig. S2). The smallest AuNPs (2 nm) show no absorption peak in the visible region since the small diameter of the nanoparticles prevent surface plasmon modes^42^.

### Antibacterial Surface Characterisation

Typically, swell-encapsulation of aqueous AuNPs is performed in acetone-water mixtures^34^. However, in order to achieve better swelling, organic solvents have been proposed^16^. The synthesis of AuNPs in the organic phase was favorable for the swell-encapsulation process used to encapsulate AuNPs into polyurethane. Information on how the AuNP solution concentrations were prepared can be found in the experimental section. AuNPs of 2 and 5 nm diameter were swell-encapsulated into PU using toluene and for the 3 nm AuNPs, a mix of hexane and dichloromethane (DCM) was used as the swelling agent. Hexane/DCM was required for the 3 nm sized AuNP since these particles were dispersible in hexane but not in toluene, where they instead assemble at the air-liquid interface^40^. The incorporation of organic solvents facilitates diffusion of the AuNPs through the swollen polyurethane, after which they are immobilised within as the solvent evaporates and the polymer shrinks back to its original size. This swelling technique opens up the polymer structure enhancing the incorporation of AuNPs. AuNPs were swell-encapsulated at both 0.1 mg mL^−1^ and 1 mg mL^−1^ solution concentrations to investigate the effect of low and high-incorporated densities of nanoparticles on bactericidal activity. The AuNP-incorporated polymers were subsequently coated with CV by dipping the polymer samples into an aqueous CV solution (1 mM) for 72 hours. To ensure sufficient uptake of the AuNPs was achieved, the surfaces were characterised using UV-visible absorption spectroscopy, XPS, ToF-SIMS, and optical/fluorescence microscopy. PU samples swell-encapsulated with AuNPs and coated with CV are subsequently labeled as PU-AuNPs-CV.

**Figure 2:**
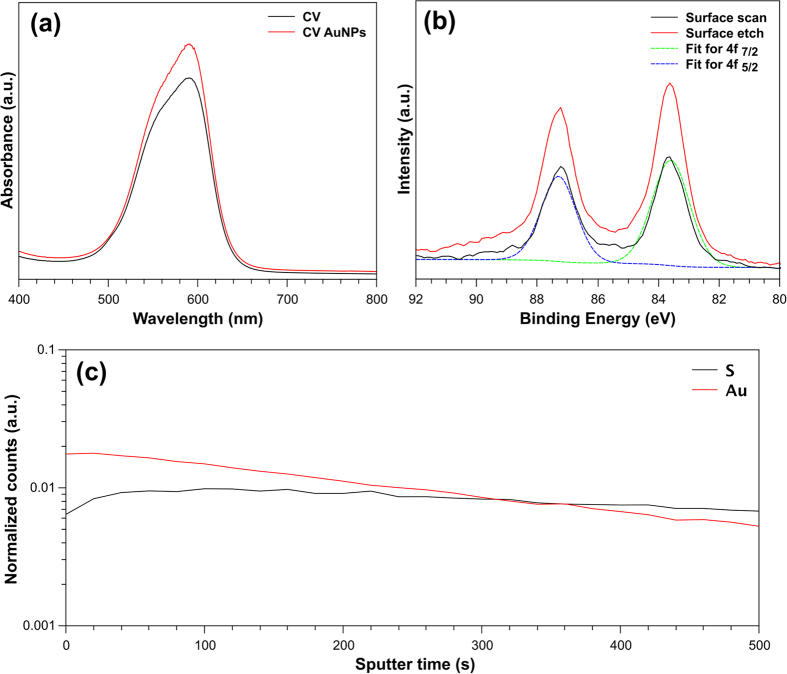
(**a**) UV-visible absorption spectra for AuNP, PU-CV and PU-AuNPs-CV (all using 2 nm AuNPs). (**b**) Represents the XPS for PU-AuNPs-CV samples where the black line shows the surface scan and the red line shows the surface etch (depth profile). For the depth profile, the sample was etched for 200 seconds. The green line represents the fit for Au 4f _7/2_ (FWHM: 1.4 eV) and the blue line represents the fit for Au 4f_5/2_ (FWHM: 1.4 eV). (**c**) ToF-SIMS depth profile for PU swell encapsulated with AuNPs. The ToF-SIMS was acquired using a 500 V Ar + beam for sputtering.

Figure 2 shows the UV-visible absorption and XPS spectroscopy for the CV coated PU (PU-CV) and PU-AuNPs-CV encapsulated with 2 nm AuNPs. The UV-visible absorption spectra shows that the PU-CV, and PU-AuNPs-CV samples had an absorption peak at 590 nm with a shoulder at 550 nm, which is characteristic of CV^16^,^43^. While 2 nm sized AuNPs do not show an absorption peak (for reasons mentioned above), the PU-AuNPs-CV samples show slightly higher absorption intensity at 590 nm when compared to the PU-CV sample. Since the absorption intensity is not significantly higher, the small absorption enhancement cannot be attributed to the AuNP incorporation and is within the experimental error. To explore the effect of concentration and immersion time of CV in polyurethane, further UV-Vis absorbance measurements were performed. These measurements showed that while the uptake of CV increased with concentration and immersion time, concentrations too high resulted in aggregation of the dye (Supplementary Figs S3 and S4). Therefore, the optimised conditions for PU were a CV concentration of 1 mM and coating for 72 hours.

XPS analysis showed peaks in the carbon, nitrogen, and oxygen regions on all the surfaces analysed (AuNP encapsulated and control) and correlate to the PU substrate. For samples encapsulated with 2 nm AuNPs, XPS analysis showed a typical Au 4 f doublet at binding energies 83.6 eV (4 f _7/2_) and 87.3 eV (4 f _5/2_) indicative of a spin-orbital split of 3.7 eV^44^. A 200 second surface etch was performed to confirm AuNPs had penetrated the surface. This involved obtaining a monatomic depth profile of the PU-AuNPs-CV samples using an ion beam to etch layers of the surface revealing subsurface information. The depth profile corresponded to a penetration of roughly 50 nm for each PU-AuNPs-CV sample. The increased number of counts in the XPS spectra suggests that AuNPs in the bulk, are well embedded into the polyurethane. XPS analysis on 3 and 5 nm AuNPs encapsulated in PU showed peaks in the 4 f _7/2_ region at 83.7 eV and 83.3 eV, respectively, consistent with a majority of the sample being Au^0 ^44. Peaks in the 4 f _5/2_ region for 3 and 5 nm AuNPs were 87.4 eV and 87.0 eV respectively. The spin orbital splitting of Au 4 f peaks for both 3 and 5 nm AuNPs was 3.7 eV. The XPS spectra for 3 and 5 nm AuNPs encapsulated in PU can be seen in Supplementary Fig. S5.

To further characterise the AuNPs on the surface of the polyurethane, ToF-SIMS was performed on the AuNPs and CV-AuNPs samples. Negative SIMS confirmed the presence of Au in both the CV-AuNPs and AuNPs-only PU samples (m/z = ~197 amu, Supplementary Fig. S6). Figure 2(c) shows the ToF-SIMS depth profile for AuNPs containing PU that shows sulfur (S) from DDT and gold (Au) to have consistent ionization efficiencies across the depth of the sample. There was no increase in secondary ion yield of the AuNPs with depth. While XPS showed an increase in Au intensity after a 200 second etch which corresponded to a depth of approximately 50 nm (calibrated), ToF-SIMS is a more surface sensitive technique, which means the sampling depth is considerably less^45^,^46^. A depth profile of CV-AuNP PU was also acquired. Supplementary Figure S7 confirms the presence of S, Au and chlorine (Cl) where the Cl is expected due to the CV coating. Therefore, XPS confirms the presence of AuNPs in both the bulk and the surface whereas ToF-SIMS confirms the presence on the topmost surface layers.

It has been suggested that for the most photoactive bactericidal surfaces, high surface concentrations of the photosensitised dye are beneficial^16^,^36^. This is because the photo-generated cytotoxic species such as ROS have nanoscale diffusion distances^43^. Since the diffusion distances are short, only ROS generated very close to bacteria will kill them.

**Figure 3:**
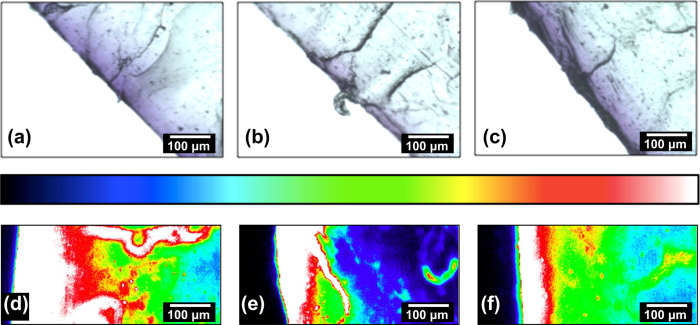
Optical microscope images of 6 μm thick cross sections of (**a**) PU-AuNPs-CV sample containing 2 nm AuNPs, **(b)** PU-AuNPs-CV sample containing 3 nm AuNPs, and (**c**) PU-AuNPs-CV sample containing 5 nm AuNPs. The polymer section imaged is positioned at an incline on the upper-right hand corner. CCD falsecolored fluorescence microscopy images of the PU samples imaged in (**d**–**f**). (**d**) PU-AuNPs-CV sample containing 2 nm AuNPs, (**e**) PU-AuNPs-CV sample containing 3 nm AuNPs, and (**f**) PU-AuNPs-CV sample containing 5 nm AuNPs. Note that the fluorescence intensity scale shown on top increases from black (correlating to no fluorescence), through to white (correlating to high fluorescence intensity).

The distribution of the CV dye in the PU-AuNPs-CV samples containing 2, 3 or 5 nm AuNPs was analysed using light microscopy (Fig. 3a–c, respectively). The samples were embedded in wax and cut using a microtome, such that the sample thickness was 6 μm. All three PU-AuNPs-CV samples (Fig. 3a–c) showed preferential surface localization of the dye, due to simple diffusion into the polymer, as water is a non-swelling solvent for PU. In addition, the light microscope images suggest that the dye diffused further in the 2 nm AuNP (Fig. 3a) 5 nm AuNP (Fig. 3c) encapsulated samples whereas the dye diffusion gradient was smaller in the 3 nm AuNP encapsulated sample (Fig. 3b). We speculate that this is due to the 3 nm AuNP encapsulation using a hexane/DCM mixture rather than toluene like the 2 and 5 nm AuNPs encapsulations, and somehow affecting the diffusion rate of CV through the polymer.

The PU-AuNPs-CV samples were also analysed using fluorescence microscopy, to provide a clear visual comparison of the uptake efficiency of the dye and the extent of dye diffusion, across the sample range. The CV fluorescence was monitored at 630 nm and the resultant false-colored images are shown in Fig. 3(d–f). The fluorescence images show one half of the polymer section examined, providing an overview of the dye diffusion gradient through the polymer bulk. The false color scale ranges from black - corresponding to areas where fluorescence is low and thus no dye is present - through to white - corresponding to high fluorescence and accordingly, high CV concentrations. The PU-AuNPs-CV samples showed surface localization of the dye, represented by a white band at the polymer edge. It was found that the PU-AuNPs-CV sample containing 2 nm AuNPs had a slightly wider intense fluorescence band at the polymer surface than the 3 nm and 5 nm AuNP encapsulated samples. It can be speculated that the small size of the 2 nm AuNPs may not inhibit CV diffusion through the polymer to the same extent as the 3 nm or 5 nm samples. While the optical and fluorescence microscopy is merely a semi qualitative analysis, the intense fluorescence band (shown in white false color on Fig. 3(d)), indicates that more CV is present in the 2 nm PU-AuNPs-CV sample. Nevertheless a more quantitative analysis of the dye uptake is required.

Previous studies have shown hydrophobicity plays an important role in bacterial adhesion^47^; therefore large variations in water contact angles between samples will have a significant effect on the adhesion of bacteria. To investigate the wetting properties for the different samples, water contact angle measurements were performed (Table 1). While there were slight variations in water contact angles, exposure to different solvents and the incorporation of CV and AuNPs (of various sizes) did not result in any significant differences in wetting properties. The slightly lower contact angle for the CV hexane and DCM sample may be due to a change in surface morphology. Since the variation of contact angle does not differ significantly, the likelihood that the samples will differentially affect bacterial adhesion is low.

In order to investigate the stability of the CV coated samples in phosphate buffer saline (PBS) solution, the concentration of CV that leached into solution was measured using UV-Vis (Supplementary Fig. S8). The results showed that all samples initially released a low concentration of CV into PBS solution and the leaching effects plateaued rapidly with time. The initial leaching of the CV into PBS solution can be attributed to loosely bound dye molecules on the antibacterial substrate. Over a period of 300 hours, the leaching concentrations for PU-CV and PU-AuNPs-CV samples were all less than 3 × 10^−6^ M. Importantly, CV is a low-toxicity photosensitiser which is within the therapeutic window in which you can kill bacteria without resulting in irreversible damage to mammalian cells^36^. Therefore, small concentrations of CV that leached into the PBS solution should not result in adverse effects in future clinical applications of these materials.

In addition to the studies above, photostability testing of PU-CV and PU-AuNPs-CV encapsulated with 2, 3 or 5 nm sized AuNPs samples were investigated. In doing so, the samples were exposed to white hospital light and their photodegradation was measured over a period of 30 days using UV-Vis (Supplementary Fig. S9). The photodegradation tests indicated that PU-AuNPs-CV samples encapsulated with 2 or 3 nm sized AuNPs were the least effected after 30 days of irradiation (~6,000 lux) measuring 32% and 18% degradation, respectively. Notably, light intensities in hospital wards and corridors have been previously reported at ~200 lux, 30 times lower than the 6,000 lux light intensity which we investigate^11^,^48^. While light intensities are higher in operating theatres, our calculated photodegradation rates suggest that the PU-AuNPs-CV samples encapsulated with 2 or 3 nm sized AuNPs would demonstrate antibacterial potency for several years if used in hospital wards or corridors. Further studies are required in order to determine the relationship between AuNP size and photodegradation.

### Microbiological Analysis

**Figure 4:**
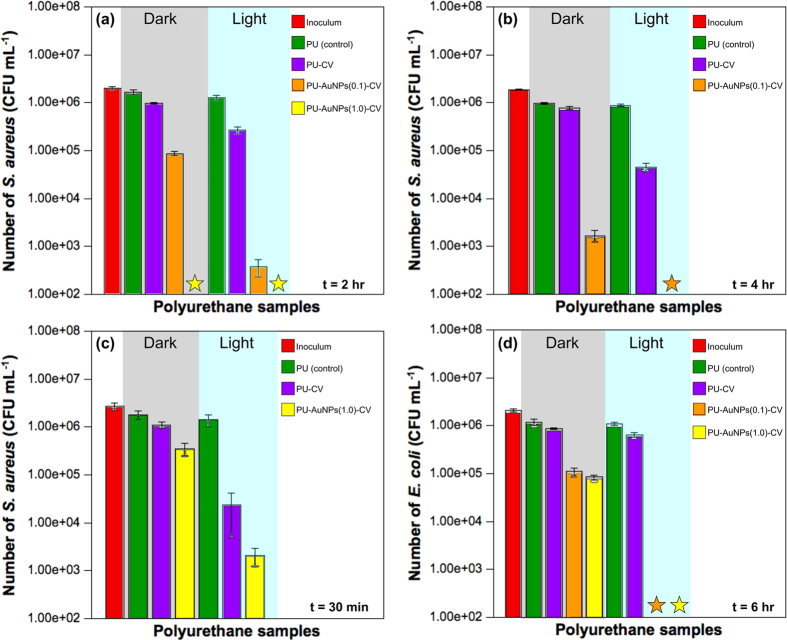
(**a**) 0.1 mg mL^−1^ and 1 mg mL^−1^ 2 nm AuNP encapsulated PU (2 hours, *S. aureus*), (**b**) 0.1 mg mL^−1^ 2 nm AuNP encapsulated PU (4 hours, *S. aureus*), (**c**) 1 mg mL^−1^ 2 nm AuNP encapsulated PU (30 minutes, *S. aureus*), (**d**) 0.1 mg mL^−1^ and 1 mg mL^−1^ 2 nm AuNP encapsulated PU (6 hours, *E. coli*). The stars represent kills below the detection limit (>log 4).

The antibacterial activity of the modified samples was tested against *S. aureus* and *E. coli* as representative Gram-positive and Gram-negative bacteria, respectively. All samples were prepared using the same method with prior swell-encapsulation of the solvent and AuNPs before coating with CV. To ensure that any antibacterial activity of the modified samples was not due to the capping agent used to synthesise the nanoparticles, DDT-encapsulated PU was tested against both bacteria and showed no statistically significant antibacterial activity. The AuNP-encapsulated PU also showed no bactericidal activity under the experimental conditions used (dark and light) and this data can be found online in Supplementary Fig. S10. It was only when coupled with CV, that AuNPs exhibited antibacterial activity. PU coupled with CV only (PU-CV) also showed some bactericidal activity as reported in previous studies^16^. Figure 4 presents the results of the antibacterial tests carried out against *S. aureus* and *E. coli* for PU samples swell-encapsulated with 2 nm AuNPs coated in CV (PU-AuNPs-CV). For PU swell-encapsulated with 1 mg mL^−1^ and 0.1 mg mL^−1^ of AuNPs, samples are referred to as PU-AuNPs(1.0)-CV and PU-AuNPs(0.1)-CV, respectively.

Figure 4(a) shows the bactericidal effects of the PU-CV and PU-AuNPs-CV samples at both low (0.1 mg mL^−1^) and high (1 mg mL^−1^) concentrations against *S. aureus,* after 2 hours of white light exposure and in the dark. Under dark conditions, no statistically significant bactericidal activity was demonstrated by the PU-CV sample, however, the PU-AuNPs(0.1)-CV samples resulted in a ~1.2 log (*P* < 0.001) reduction in the numbers of *S. aureus*. After 2 hours of white light activation (~6,000 lux), the PU-CV samples achieved a ~0.50 log (*P* < 0.001) reduction of *S. aureus* numbers, and a significant enhancement in bactericidal activity was observed for the PU-AuNPs(0.1)-CV samples containing the low concentration 2 nm AuNPs (3.5 log, *P* < 0.001). The efficacy of bacterial kill was observed to be concentration dependent. Increasing the swell encapsulation concentration of AuNPs by an order of magnitude (to 1 mg mL^−1^) showed more efficacious antibacterial activity. PU-AuNPs(1.0)-CV samples prepared by immersion in a 1.0 mg mL^−1^ 2 nm AuNP solution (one order of magnitude more concentrated) showed more efficacious bactericidal activity, reducing bacterial numbers to below the detection limit under both light and dark conditions, within 2 hours (≥4 log, *P* < 0.001). In contrast, the PU-AuNPs(0.1)-CV samples reduced the bacterial counts to below the detection limit only after 4 hours illumination when tested against *S. aureus*, with a 2.8 log reduction in bacterial numbers in the dark (Fig. 4(b)).

Since the PU-AuNPs(1.0)-CV samples reduced the numbers of *S. aureus* to below the detection limit in 2 hours, these samples were subjected to a 30 minute test to investigate their effectiveness on a shorter time scale (Fig. 4(c)). The PU-CV samples showed no significant reduction in bacterial numbers in the dark. However, upon irradiation with white light, the PU-CV samples induced photosensitisation, achieving a 1.8 log reduction in *S. aureus* numbers within 30 minutes. The PU-AuNPs(1.0)-CV samples showed limited antibacterial activity under dark conditions with a reduction in bacterial numbers of ~0.5 log (*P* < 0.001). In the light, the PU-AuNPs(1.0)-CV samples exhibited enhanced antibacterial activity, resulting in a 3.0 log (*P* < 0.001) reduction in the numbers of *S. aureus* in 30 min.

PU-AuNPs(1.0)-CV samples that were swell encapsulated with 3 or 5 nm sized AuNPs were also tested against *S. aureus*. The results showed that the antibacterial activity of the samples encapsulated with 3 or 5 nm sized AuNPs was less than that exhibited by the materials containing the 2 nm sized AuNPs (Table S1). This is consistent with previous reports for aqueous AuNPs which were encapsulated into MB coated silicone^34^. Under dark conditions, PU-AuNPs(1.0)-CV samples encapsulated with 3 or 5 nm AuNPs achieved a 2.0 log and 0.75 log reduction, respectively, in the numbers of *S. aureus* in 2 hours. After 2 hours of irradiation, the PU-AuNPs(1.0)-CV samples encapsulated with 3 or 5 nm AuNPs achieved a 3.0 log and 3.5 log reduction, respectively, in the numbers of *S. aureus* (Supplementary Table S1). Previous work has reported that AuNPs which are 3 nm or less in diameter have a significant difference in chemical reactivity when compared to organogold complexes^49^ and larger gold nanoparticles^50^. In addition to these reports, this trend in decreasing antibacterial activity with AuNP size is consistent with our previous work^34^.

Each of the samples were then tested against *E. coli*, however, longer incubation times were required due to the reduced susceptibility of Gram-negative bacteria to photodynamic therapy. The PU-CV samples showed poor antibacterial activity when exposed to *E. coli* for 3 and 6 hours in the dark. Under dark conditions, PU-AuNPs-CV treated with either 0.1 and 1.0 mg mL^−1^ 2 nm AuNP solutions resulted in a reduction in bacterial numbers of ~1.2 log (*P* < 0.001) within 6 hours (Fig. 4(d)). By activating the synergistic photobactericidal effect with white light irradiation, the PU-AuNPs-CV samples showed an increase in light activated antibacterial activity, reducing bacterial numbers by 2.2 log (0.1 mg mL^−1^, AuNPs) and 2.0 log (1 mg mL^−1^, AuNPs), within 3 hours (Supplementary Fig. S11) and to below the detection limit within 6 hours (Fig. 4(d)).

The PU-AuNPs-CV samples that were encapsulated with 3 or 5 nm sized AuNPs were less effective against *E. coli* when compared with the samples encapsulated with 2 nm sized AuNPs (Table S2). Under dark conditions, no significant antibacterial activity was observed for any of the samples.

After 5 hours of light conditions, the PU-AuNPs-CV samples encapsulated with 2, 3 or 5 nm sized AuNPs resulted in a reduction in *E. coli* numbers of >4.0 log, 3.1 log and 2.9 log, respectively (Supplementary Table S2).

The different susceptibilities of the Gram-positive and Gram-negative bacteria can be attributed to the differences in cell wall structure. Gram-positive bacteria have a single cell membrane with a thick, relatively porous layer of peptidoglycan and lipoteichoic acid whereas Gram-negative bacteria have a more complex wall structure comprising both an inner and outer membrane. This double membrane forms a more effective barrier between the cell and the surrounding environment^48^ rendering Gram-negative bacteria such as *E. coli* more difficult to kill than Gram-positive bacteria like *S. aureus*.

All modified PU samples demonstrate antibacterial efficacy to various extents against both Gram-positive and Gram-negative bacteria. We acknowledge however that the bactericidal assays were carried out in PBS and further experiments are required to determine the activity of the new polymers in the presence of organic matter to more closely simulate physiological fluids.

It is well known that CV poses a low toxicity risk and has previously been used as a medical antiseptic^51^. Furthermore, clinical trials have also investigated the efficiency of CV as a potential treatment against methicillin-resistant *Staphylococcus aureus* (MRSA)^52^,^53^. We speculate that the 2 nm AuNPs are the most effective against both types of bacteria due to the differing photonics of the smaller AuNPs providing a favorable pathway for the generation of reactive oxygen species which are toxic to bacteria^34^,^36^. TR-EPR from our previous study shows that the enhancement of photo-activity of MB^19^ and CV^43^ was attributed to an increase in dye triplet state when 2 nm AuNPs were present. Supplementary Figure S12 represents the Jablonski diagram and accompanying photochemical process subsequent to photoexcitation of a photosensitiser^54^. The results from the optical and fluorescent microscopy also suggests that the smaller size also allows for more AuNPs to be taken up into the PU and thus, when combined with CV, a more effective catalyst for antibacterial surfaces is created. While the light activated mechanism for the combination of AuNPs with CV in polymeric surfaces has been outlined in our previous work^36^,^43^,^54^, we postulate the mechanism behind the dark kills is related to the redox processes involving surface trap states that can undergo an electron transfer interaction with crystal violet to generate reactive oxygen species. This is the first time we have observed such a significant reduction in bacterial numbers in the dark and while it is very promising, further work is required to fully understand the mechanism behind this.

Although we have seen similar trends in our previous work with 2 nm aqueous AuNPs encapsulated into MB-coated silicone^34^, this work shows >99.99% reduction in bacterial numbers using white light (rather than laser irradiation) from size-controlled thiol-capped AuNPs encapsulated into CV-coated PU. Without requiring more intense laser irradiation, this work shows a simple and straightforward approach to swell-encapsulate AuNPs into PU and effectively kill bacteria under conditions of much lower light intensities than previous reports. This work also demonstrates the importance of particle size, whereby non-plasmonic particles (<3 nm) are far more effective, where the use of thiol-capped organic soluble AuNPs enables higher loadings into PU-CV.

## Conclusion

Healthcare-associated infections that are linked to resistant strains of bacteria are a worldwide concern and the development of antibacterial coatings can play a crucial role in reducing these threats. The swell-encapsulation technique is a straightforward and up-scalable approach to incorporate AuNPs with a photosensitised in PU to kill harmful bacteria. Size controlled (2, 3 or 5 nm) thiol-capped AuNPs have been successfully demonstrated to enhance the antibacterial activity when encapsulated into PU-CV surfaces. We also demonstrate that the presence of the capping agent, dodecanethiol, does not alter the antibacterial properties. This means that the thiol is not intrinsically antimicrobial and its incorporation aids in the synthesis of small, size controlled AuNPs and their incorporation into PU. 2 nm AuNPs had superior antibacterial activity when compared to 3 or 5 nm AuNPs encapsulated into PU-CV surfaces. We postulate that this is due to the differing photonic properties of the smaller AuNPs and better diffusion into the swelled PU material. 1 mg mL^−1^ and 0.1 mg mL^−1^ swell encapsulation concentrations of AuNPs were also studied whereby samples encapsulated with 1 mg mL^−1^ AuNPs were found to be the more effective against both Gram-positive (*S. aureus*) and Gram-negative bacteria (*E. coli*) under dark and light conditions. We show that there is a clear size and concentration effect on the AuNPs, which heavily influences the antibacterial activity. In contrast to our previous work, which used aqueous AuNPs, MB-dye as a sensitised, intense lasers as a light source and silicone as a polymer, for the first time, we show reduction below the detection limit (>4 log) of *S. aureus* under both dark and light conditions and of *E. coli* under white light conditions. AuNPs suspended in organic solvents allow for precise size control as well as ease of polymer incorporation *via* the swell encapsulation technique. This study demonstrates optimised conditions for the preparation of antibacterial surfaces to be used in healthcare environments. In addition to our previous research, these new findings show that this approach is effective under multiple conditions, which can be tailored to suit hospital or healthcare environments. This opens more possibilities for different technology and new applications in antibacterial coatings.

## Methods

### Nanoparticle Synthesis

#### 2 nm AuNP

2 nm AuNPs were prepared using a method adapted from Brust *et al*.^39^. Deionized (DI) water (resistivity 15 MΩcm) was used in all experiments. An aqueous solution of chloroauric acid trihydrate (VWR, UK) (6 mL, 30 mM) was mixed with tetraoctylammonium bromide (Sigma Aldrich, UK) (16 mL, 50 mM) in toluene (Fisher Scientific, UK). The mixture was vigorously stirred until the gold salt was completely transferred to the organic phase and 1-dodecanethiol (DDT) (Sigma Aldrich) (34 mg) was added. Freshly, prepared aqueous sodium borohydride (VWR, UK) (5 mL, 0.4 mM) was added dropwise and the solution was continuously stirred over a period of 3 h. The organic layer was subsequently extracted and evaporated to 2 mL. Ethanol (80 mL) was added to the organic layer and stored for 4 h at −18 °C, after which the black precipitate was filtered off and washed with ethanol (Merck Millipore, UK). The freshly made nanoparticles were subsequently suspended in toluene for further use.

#### 3 nm AuNP

3 nm AuNPs were prepared using a method adapted from Martin *et al*.^40^. An aqueous solution of sodium borohydride in sodium hydroxide (750 μL, 50 mM) was added to an aqueous solution of chloroauric acid trihydrate (VWR, UK) (250 μL, 50 mM) in hydrochloric acid (Merck Millipore), and the subsequent solution was vortexed to ensure the solutions were thoroughly mixed. Acetone (Sigma Aldrich, UK) (12.5 g) was added to the mixture and stirred, after which hexane (Sigma Aldrich, UK) (12.5 g) and DDT (1 μL) were added with further stirring. The burgundy colored AuNP/hexane layer was then extracted. It has been demonstrated that the nanoparticles are stable in hexane but not toluene^40^.

#### 5 nm AuNP

Synthesis of 6 nm AuNPs was based on the method from Palgrave *et al*.^41^ which is similar to Brust’s synthesis. The AuNPs were formed using a two-phase chemical reduction method, but without the presence of DDT. An aqueous solution of chloroauric acid trihydrate (VWR, UK) (15 mL, 28 mM) was mixed with tetraoctylammonium bromide (40 mL, 50 mM) in toluene. The mixture was vigorously stirred until the gold salt was completely transferred to the organic phase. Freshly made aqueous sodium borohydride (VWR) (5 mL, 0.2 mM) was then added dropwise with continuous stirring over a period of 30 min period. The red organic layer was subsequently extracted and washed with diluted sulfuric acid (50 mL) three times. The organic layer was dried with magnesium sulfate. The nanoparticles were subsequently suspended in toluene for stability forming a lighter red color.

### Nanoparticle swell-encapsulation

#### 2 and 5 nm AuNP swell-encapsulation

Medical grade PU (Branford, CT, USA) was cut into 1 × 1 cm squares. 2 and 5 nm AuNPs were swell-encapsulated into the PU by immersing the squares into the toluene dispersed AuNPs. AuNP swelling solutions were made up to 0.1 and 1.0 mg mL^−1^ and the PU squares were swell encapsulated in the dark for 48 h. After 48 h, the swolen samples were rinsed with toluene to remove residual AuNPs from the surface and left to dry where they shrink back to their original size.

#### 3 nm AuNP swell-encapsulation

3 nm AuNPs were swell encapsulated using the same parameters as above however the swelling procedure was carried out using a mixture of hexane and dichloromethane with a ratio of 5.5:4.5. This ratio was selected as it resulted in swelling almost identical to that of the samples swollen in toluene.

### Dye coating on Polyurethane

AuNP encapsulated PU and bare PU samples were coated with CV by immersing the 1 × 1 cm squares into a 1 mM solutions of CV for 72 h in the dark. After 72 h, the samples were rinsed with DI water to remove residual dye molecules from the surface. The samples were left to dry before being used further.

### Characterisation of Dye Uptake

A Perkin-Elmer Lambda 25 UV-Vis spectrometer was used to record the UV-Vis absorption spectra of the modified PU samples within the range 400–800 nm and the corresponding AuNP solutions used to synthesis these polymers (200–800 nm). The CV distribution within cross-sections of PU samples was analysed using optical and fluorescence microscopy. Control and modified PU samples were embedded vertically in paraffin blocks. 6 μm sections were sliced using a microtome (Leica RM2235). These 6 μm sections of each sample were imaged using a light microscope (Olympus UK Ltd., model BH2) with a colored CCD digital camera (Lumenera, model Infinity 1) to obtained magnified images. Subsequently fluorescence images of same samples were obtained for the purpose of comparison. The fluorescence microscope (Olympus UK Ltd., Model IMT-2) was used with a cooled CCD camera (Princeton Instruments Ltd., Model PIXIS 512). A laser with excitation wavelength of 532 nm was used to excite CV fluorescence, which was detected by a bandpass filter (Omega Optical Inc., model 640DF30) centered at 640 nm. The images were subsequently analysed using Roper Scientific Software WinSpec/32.

### Dye Leaching and Photostability

The stability of PU-CV and PU-AuNPs-CV in phosphate buffer saline (PBS, Dulbecco A) (OXOID) at room temperature was investigated. PU-CV and PU-AuNPs-CV samples (1 cm^2^) were immersed in PBS (2.5 mL) for an extended time period and the UV-Vis absorbance of the PBS (590 nm, Pharmacia Biotech Ultrospec 2000) was measured periodically to monitor any leaching of CV from the polymer into PBS. A calibration curve of CV with the absorbance at 590 nm was generated in order to determine the concentration of CV that was leached from the samples into PBS.

Control and modified samples were placed under white light in a box for an extended duration up to a month. A white light source (General Electric 28 W Watt Miser™T5 2D compact fluorescent lamp) emitted an average light intensity of 2650 ± 50 lux at a distance of 30 cm from the samples. At each time period samples were investigated by using the same UV-Vis Spectrometer as above.

### Characterisation of Nanoparticles and Antibacterial Surfaces

Transmission electron microscopy (TEM) was used to analyse the size of the synthesised AuNPs. Samples were prepared by drop-casting the respective AuNP suspensions onto holey carbon-coated copper grids and drying in air overnight. TEM micrographs were collected using a JEOL 2010 TEM operating at 200 kV. Image collection and processing was performed with Gatan Digital Micrograph software. Particle size analysis was carried out using ImageJ software.

X-ray photoelectron spectroscopy (XPS) was carried out using a Thermo Scientific K-alpha photoelectron spectrometer with monochromatic Al-K_α_ radiation to analyse the uptake of the different sized AuNPs in PU. Higher resolution scans were obtained for the principal peaks of Au (4 f), N (1 s), O (1 s) and C (1 s) at a pass energy of 50 eV whereas survey scans were gathered in the range 0–1100 eV (binding energy) with a pass energy of 160 eV. Peak positions were calibrated to carbon (285 eV) and plotted using the CasaXPS and qtiplot software.

Time-of-Flight Secondary Ion Mass Spectrometry (ToF-SIMS) was used to perform elemental depth profiles on CV-AuNP PU surfaces. The ToF-SIMS instrument was a Physical Electronics TRIFT V NanoToF. Depth profiles were conducted using 500 V Ar + beam operating in pulsed and continuous modes for analysis and sputtering cycles, respectively.

A FTA 1000 Drop Shape Instrument was employed to measure the equilibrium water contact angle for each sample type prepared for microbiological testing. The average contact angle was measured over 10 measurements for each type of sample, using a droplet of water (5.0 μL) dispensed by gravity from a gauge 30 needle. The data was subsequently analysed using FTA32 software.

### Microbiology

1 × 1 cm PU samples were used to test the antibacterial activity of the following samples: (i) control (treated with toluene), (ii) Au NP-encapsulated polymer (PU-AuNP), (iii) crystal violet-coated polymer (PU-CV) and (iv) Au NP-encapsulated and CV-coated polymer (PU-AuNPs-CV). The antibacterial activity of these samples was tested against *Staphylococcus aureus* 8325-4 and *Escherichia coli* ATCC 25922. These organisms were stored at −70 °C in Brain-Heart-Infusion broth (BHI, Oxoid) containing 20% (v/v) glycerol and propagated onto either Mannitol Salt agar (Oxoid) in the case of *S. aureus* or MacConkey agar (Oxoid) in the case of *E. coli*, for a maximum of 2 subcultures at intervals of 2 weeks.

BHI broth was inoculated with 1 bacterial colony and cultured in air at 37 °C for 18 h with shaking at 200 rpm. The bacterial pellet was recovered by centrifugation, (20 °C, 2867.2 *g*, 5 min), washed in phosphate-buffered saline (PBS; 10 mL), centrifuged again under the same conditions, and then the bacteria were re-suspended in PBS (10 mL). The washed suspension was diluted 1000-fold to achieve an inoculum of ~10^6^ cfu/mL. In each experiment, the inoculum was confirmed by plating 10-fold serial dilutions on agar for viable counts. Triplicates of each polymer sample type were inoculated with 25 μL of the inoculum and covered with a sterile cover slip (2.2 cm^2^). The samples were then irradiated for up to 6 h using a white light source (General Electric 28 W Watt Miser^TM^ compact fluorescent lamp) emitting an average light intensity of 6,000 lux or incubated in the dark for the same irradiation time. After incubation, the inoculated samples and cover slips were added to PBS (450 μL) and mixed well using a vortex mixer. The neat suspension and 10-fold serial dilutions were plated on agar for viable counts and incubated aerobically at 37 °C for 48 h (*S. aureus*) or 24 h (*E. coli*). The experiment was repeated three times and the statistical significance of the following comparisons was analysed using the Mann-Whitney U test: (i) control (polymer only) vs. inoculum; (ii) CV vs. control, (iii) CV and Au NP vs. CV alone. All statistics reported with antibacterial activity were calculated using a two-tailed T-test.

## Additional Information

**How to cite this article**: Macdonald, T. J. *et al*. Thiol-Capped Gold Nanoparticles Swell-Encapsulated into Polyurethane as Powerful Antibacterial Surfaces Under Dark and Light Conditions. *Sci. Rep.* **6**, 39272; doi: 10.1038/srep39272 (2016).

**Publisher's note:** Springer Nature remains neutral with regard to jurisdictional claims in published maps and institutional affiliations.

## Supplementary Material

Supplementary Information

## Figures and Tables

**Table 1:** Average water contact angles on a range of PU polymers: PU (control), CV-coated (toluene), CV-coated (hexane and DCM), CV-coated, 2 nm AuNP encapsulated, CV-coated, 3 nm AuNP encapsulated and CV-coated, 5 nm AuNP encapsulated samples.

Polyurethane Sample	Water contact angle (^ο^) ± standard deviation
PU (Control)	99 ± 1.0
PU-CV toluene	101 ± 0.8
PU-CV hexane and DCM	87 ± 2.5
PU-CV + 2 nm AuNP	97 ± 1.5
PU-CV + 3 nm AuNP	102 ± 3.0
PU-CV + 5 nm AuNP	95 ± 1.1
