# Supplementary material for: Thiol-Capped Gold Nanoparticles Swell-Encapsulated into Polyurethane as Powerful Antibacterial Surfaces Under Dark and Light Conditions
Source: Sci Rep. 2016 Dec 16;6:39272. doi: 10.1038/srep39272 (PMC5159832; doi:10.1038/srep39272)
Supplement: Supplementary Information [file srep39272-s1.pdf]

Supplementary Information

Thiol-Capped Gold Nanoparticles Swell-Encapsulated into Polyurethane as Powerful Antibacterial Surfaces Under Dark and Light Conditions

*Thomas J. Macdonald, Ke Wu, Sandeep K. Sehmi, Sacha Noimark, William J. Peveler, Hendrik du Toit, Nicolas H. Voelcker, Elaine Allan, Alexander J. MacRobert, Asterios Gavriilidis and Ivan P. Parkin**

Supplementary Figures

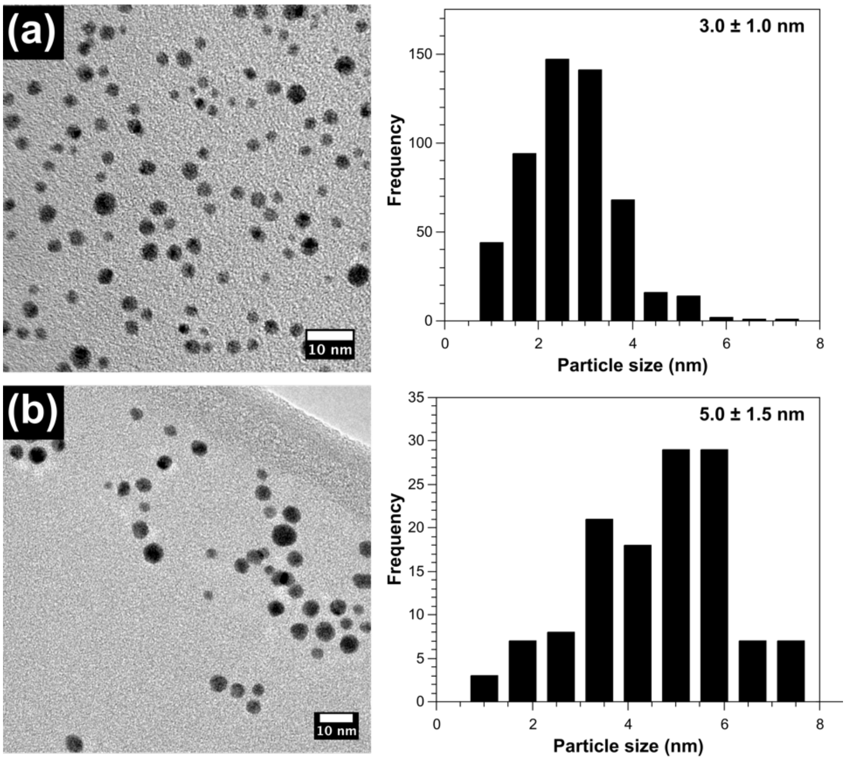

Supplementary Figure S1. (a) TEM for 3 nm (s.d. 1 nm) AuNPs with corresponding particle count (top right). (b) TEM for 5 nm (s.d. 1.5 nm) AuNPs with corresponding particle count (bottom right).

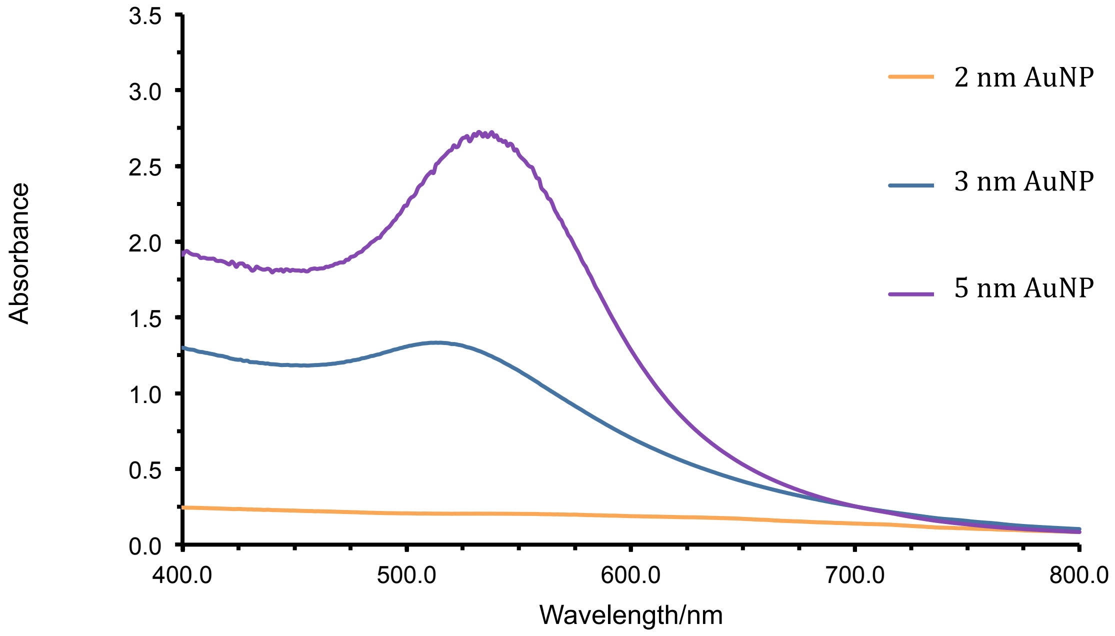

Supplementary Figure S2. Orange, blue and purple lines represent the UV-Vis spectra of 2,3 or 5 nm AuNPs, respectively. While 3 and 5 nm AuNPs represent a plasmon resonance at 517 and 532, respectively, 2 nm AuNPs do not exhibit a plasmon resonance.

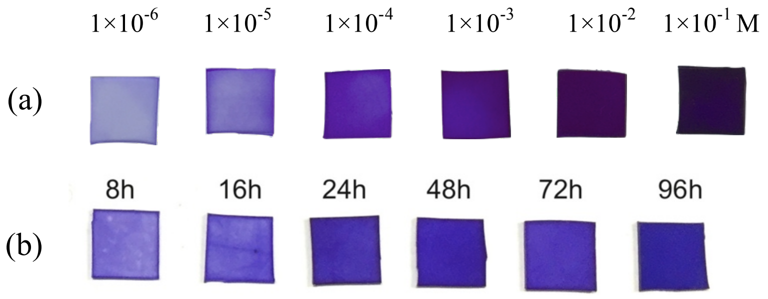

Supplementary Figure S3. PU squares prepared by immersion in: (a) aqueous CV dipping solutions of the following concentrations: 1×10^{-6} M, 1×10^{-5} M, 1×10^{-4} M, 1×10^{-3} M, 1×10^{-2} M, 1×10^{-1} M, for a period of 72 h; (b) Aqueous CV dipping solution (1×10^{-3} M) up to 96 h. The uptake of the dye increased with concentration.

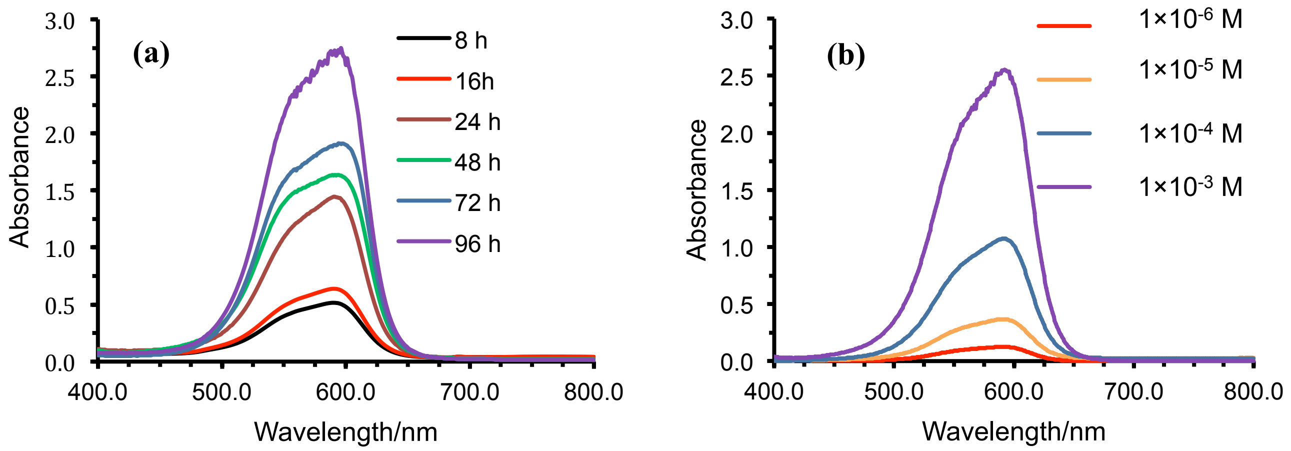

Supplementary Figure S4. UV-Vis absorbance spectra measured in the range 400–800 nm of (a) CV PU prepared by dipping in a CV solution for 72 h in various concentrations; (b) 1×10^{-3} M CV PU prepared by various dipping time up to 96 hrs. The spectra showed that the CV-coated samples had a main absorption peak at 590 nm, with a shoulder at 550 nm (**Fig. S4**). The UV−vis absorbance spectra of the PU samples immersed in CV dipping solutions with concentration $>1\times10^{-3}$ mM have absorbance intensities that exceeded the detection limit of the spectrometer and therefore, this data not shown. It can be suggested that higher concentrations of CV at the polymer surface may result in aggregation of the CV dye molecules, which can impact on the material photo-activity. Moreover, polymer squares immersed in high concentration dipping solution would lose their transparency. A solution of 1×10^{-3} mM CV was selected for further materials preparation for antibacterial testing, as it maintained relatively good transparency resulted in an efficacious uptake of the dye. However, the mechanism of the uptake of the CV dye is as yet unknown.

Using a 1×10^{-3} mM CV solution, the effects of varying the dipping time was investigated. As shown in Supplementary Fig. S3 (b) and Suppelementary Fig. S4(b), the uptake of the dye onto the polymer increased with the immersion time in the CV dipping solution, and a significant uptake of the dye occurred after 24 h along with a consistant colouration since then. PU immersed in a CV solution for 96 h showed a very strong uptake of the dye. Concerns may arise when very strong surface uptake of the dye was achieved, since it may result in greater leaching of the dye into surrounding fluids, due to an excess of loosely bound surface dye molecules. A 72 h dipping time was chosen for a strong uptake of the dye onto the PU, with a reduced risk of dye leaching into the surrounding solution.

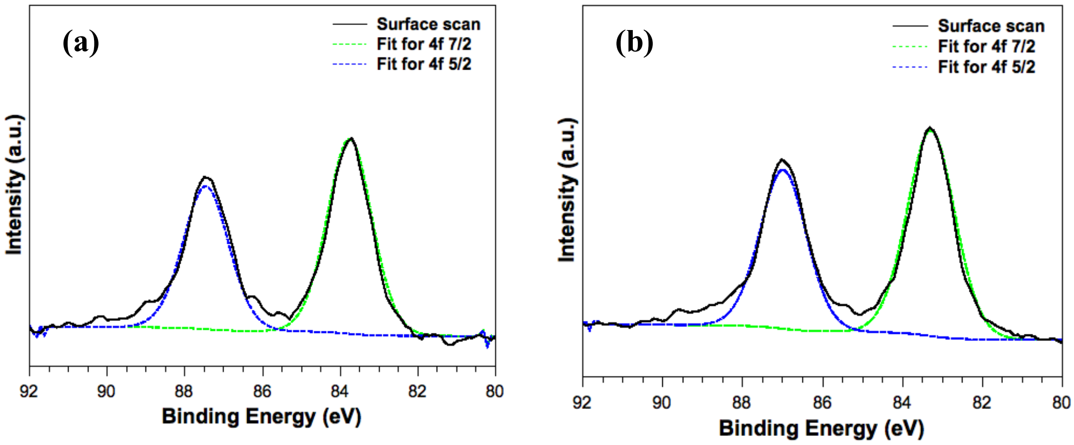

Supplementary Figure S5. XPS for Au 4f spectra for (a) 3 nm AuNPs and (b) 5 nm AuNPs.
3 nm AuNPs: 83.7 eV (4f 7/2) and 87.4 eV (4f 5/2) with a FWHM of 1.4 eV.
5 nm AuNPs: 83.3 eV (4f 7/2) and 87.0 eV (4f 5/2) with a FWHM of 1.4 eV.

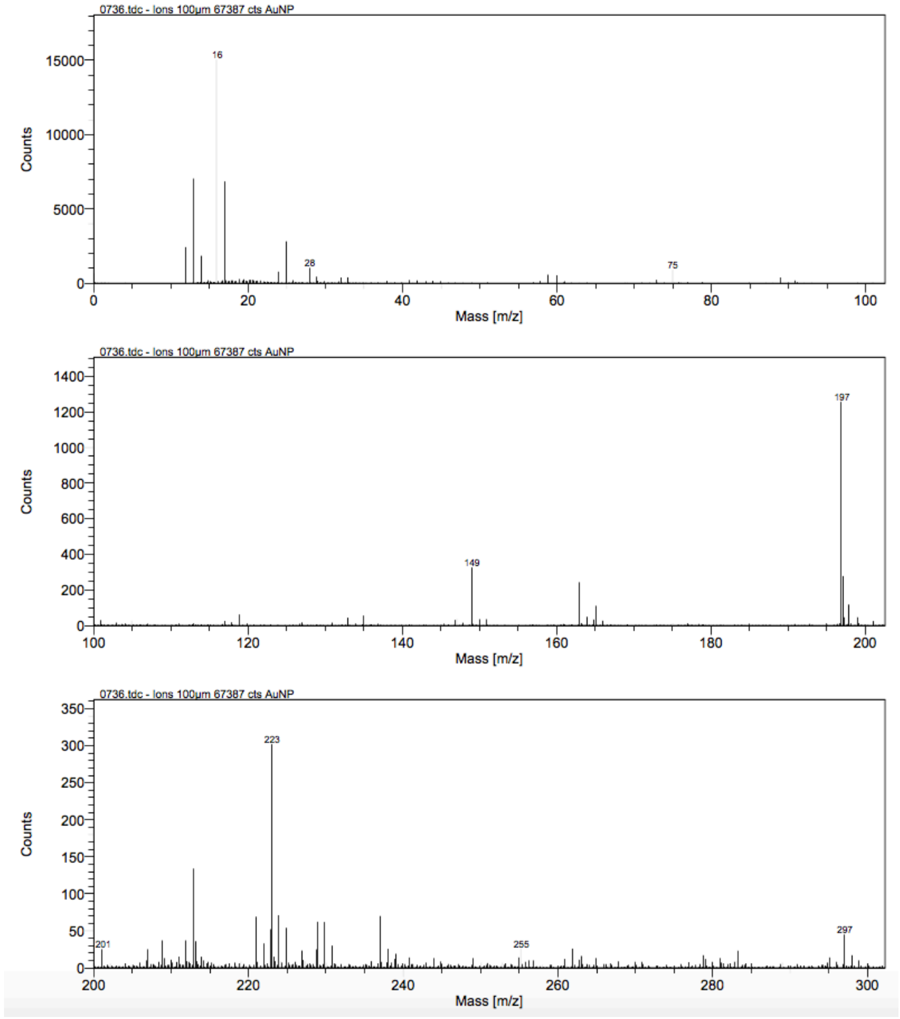

Supplementary Figure S6. Positive mode TOF-SIMS spectra for polyurthane swell-encapsulated with AuNPs. The upper, middle and bottom panels represent the mass [m/z] numbers between 0-100, 100-200 and 200-300 respectively.

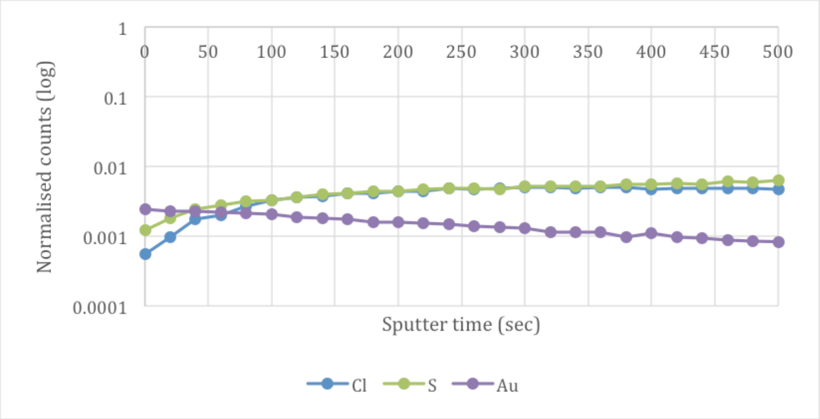

Supplementary Figure S7. ToF-SIMS depth profile for PU-AuNPs-CV samples.

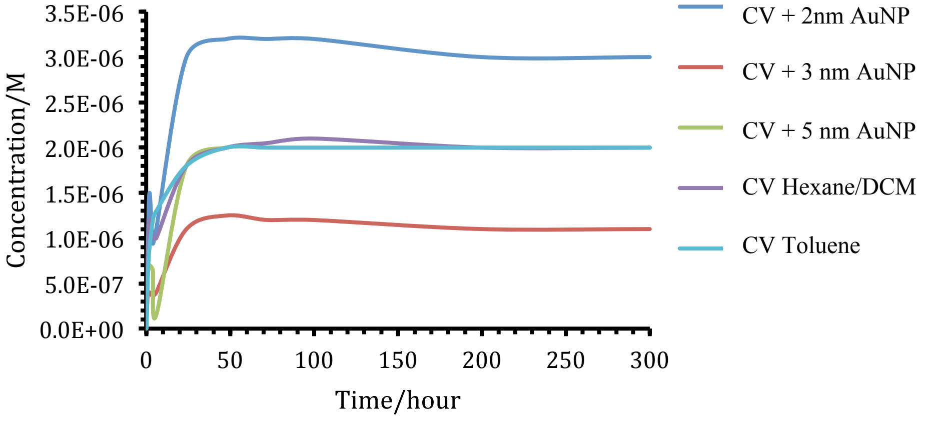

Supplementary Figure S8. Molar concentration of CV from CV-incorporated polyurethane samples into PBS solution measured as a function of time (hours) at room temperature. The release of CV from the PU-CV samples into aqueous solution (PBS) at room temperature was measured using UV-Vis. Taking the UV-Vis of the PBS solution and comparing it with a CV calibration curve measured the leaching of the CV dye. Although all samples released a low concentration of dye into solution within a short time period after immersion in the PBS, the leaching effects plateaued rapidly with time. Over a period of 300 h, the leaching of the CV dye from the samples CV 2 nm AuNP, CV 3 nm AuNP, CV 5 nm AuNP, CV (toluene) and CV (hexane and DCM) into the surrounding PBS solution, were: 3×10^{-6} M, 1×10^{-6} M, 2×10^{-6} M, 2×10^{-6} M and 2×10^{-6} M, respectively.

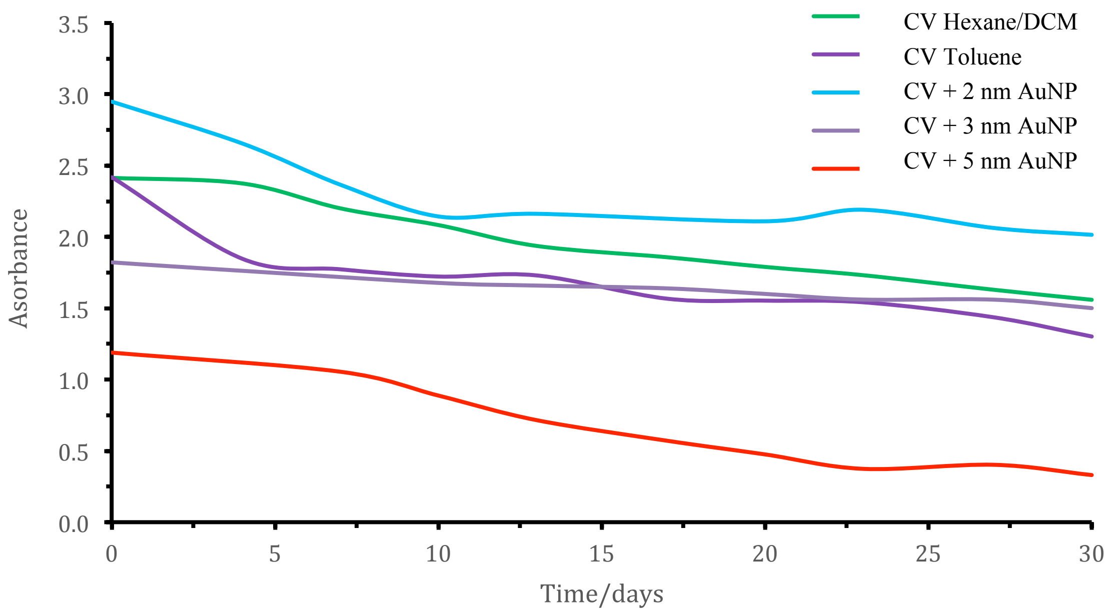

Supplementary Figure S9. Rate of photo degradation of treated polymers upon exposure to a hospital white light source (2600 lx, 30 days continuous illumination). The rate is displayed as a decrease in sample absorbance at the absorbance maximum over a period of 30 days.

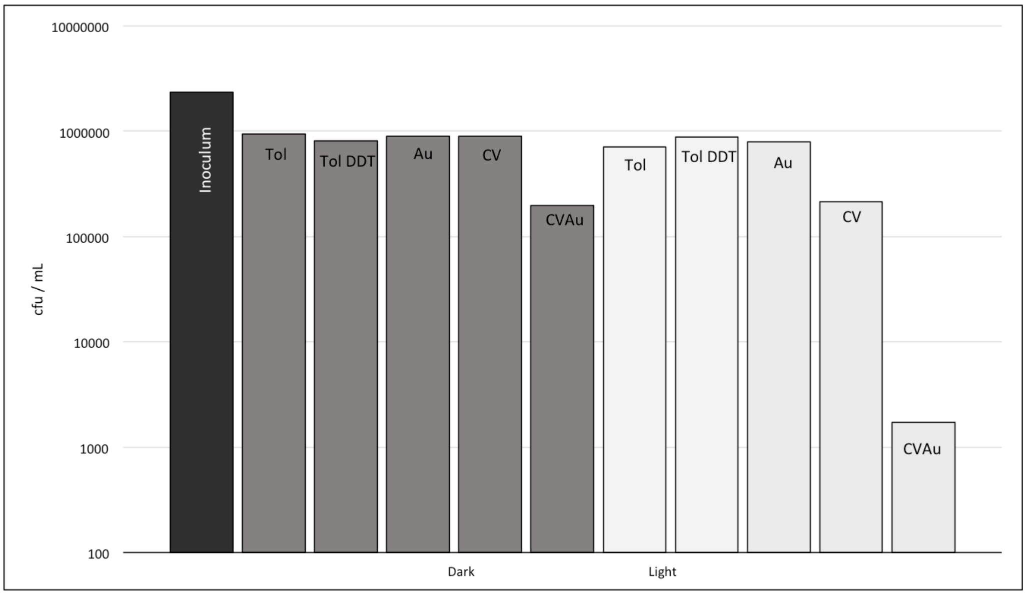

Supplementary Figure S10. Antibacterial efficacy of toluene control, AuNP control, DDT control and PU-CV and PU-AuNP-CV sample

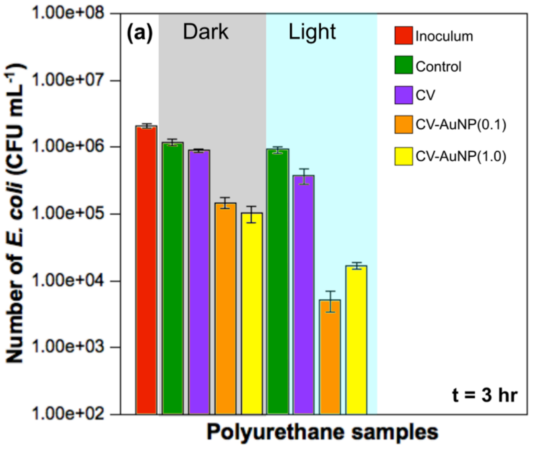

Supplementary Figure S11. Antibacterial results for PU-AuNPs-CV against *E. coli* after 3 h of dark and light conditions.

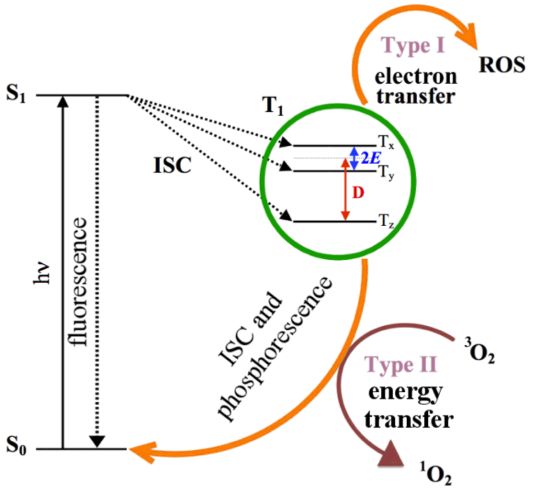

Supplementary Figure S12. Jablonski diagram and accompanying photochemical process that is subsequent to photoexcitation of a photosensitizer such as CV. Here, the ground state molecule, S_0, absorbs a photon of energy and is then promoted to an excited singlet state, known as S_1. The excited singlet state molecule can release energy radiatively via fluorescence or can undergo ISC to the excited triplet state known as T_1. The molecule that is in the triplet state can release energy as radiation via phosphorescence or transfer its energy to surrounding molecules by either a Type I or Type II photoprocess before returning to the ground state. A Type I photoprocess may be described as the transfer of electrons between the triplet state and a substrate. Whereas, a Type II photoprocess involves the quenching of the triplet state generated by molecular oxygen. Our previous studies suggest that the Type II process is favorable in this system.[1]

.

Supplementary Tables

Supplementary Table S1. Reduction in the numbers of *S. aureus* for, 3, and 5 nm PU-AuNPs-CV samples

**Dark**	**1 Hr**	**2 Hr**	**Light**	**1 Hr**	**2 Hr**
CV	0	0.2 log	CV	0.4 log	1 log
CV + 2 nm AuNP	1.3 log	>4 log	CV + 2 nm AuNP	3.3 log	>4 log
CV + 3 nm AuNP	0.6 log	2 log	CV + 3 nm AuNP	2.1 log	3.0 log
CV + 5 nm AuNP	0.3 log	0.75 log	CV + 5 AuNP	1.8 log	3.0 log

Supplementary Table S2. Reduction in the numbers of *E. coli* for CV and 2, 3, 5 nm PU-AuNPs-CV samples.

**Dark**	**5 Hr**	**Light**	**5 Hr**
CV	0	CV	2 log
CV + 2 nm AuNP	0.4 log	CV + 2 nm AuNP	>4 log
CV + 3 nm AuNP	0	CV + 3 nm AuNP	3.1 log
CV + 5 nm AuNP	0	CV + 5 AuNP	2.9 log

The above tables only highlight the results for 3 and 5 nm PU-AuNP-CV samples. All PU-AuNP-CV samples that contain 2 nm AuNPs is shown in the manuscript.

1. Noimark, S. *et al.* Comparative Study of Singlet Oxygen Production by Photosensitiser Dyes Encapsulated in Silicone: Towards Rational Design of Antimicrobial Surfaces. *Phys. Chem. Chem. Phys.* (2016). doi:10.1039/C6CP02529C
